# The Function of microRNAs in Pulmonary Embolism: Review and Research Outlook

**DOI:** 10.3389/fphar.2021.743945

**Published:** 2021-10-19

**Authors:** Mingyao Luo, Mingyuan Du, Chang Shu, Sheng Liu, Jiehua Li, Lei Zhang, Xin Li

**Affiliations:** ^1^ State Key Laboratory of Cardiovascular Diseases, Center of Vascular Surgery, Fuwai Hospital, National Center for Cardiovascular Diseases, Chinese Academy of Medical Science and Peking Union Medical College, Beijing, China; ^2^ Department of Vascular Surgery, The Second Xiangya Hospital, Central South University, Changsha, China; ^3^ The Institute of Vascular Diseases, Central South University, Changsha, China

**Keywords:** miRNA, biomarker, treatment target, pulmonary embolism, deep venous thrombosis, molecular regulation

## Abstract

Pulmonary embolism (PE) is a common pathologic condition that frequently occurs in patients with deep venous thrombosis. Severe PE may critically suppress cardiopulmonary function, thereby threatening the life of patients. Chronic pulmonary hypertension caused by PE may lead to deterioration of respiratory dysfunction, resulting in complete disability. MicroRNAs (miRNAs) are a group of abundantly expressed non-coding RNAs that exert multiple functions in regulating the transcriptome *via* post-transcriptional targeting of mRNAs. Specifically, miRNAs bind to target mRNAs in a matching mechanism between the miRNA seed sequence and mRNA 3ʹ UTR, thus modulating the transcript stability or subsequent translation activity by RNA-induced silencing complex. Current studies have reported the function of miRNAs as biomarkers of PE, revealing their mechanism, function, and targetome in venous thrombophilia. This review summarizes the literature on miRNA functions and downstream mechanisms in PE. We conclude that various related miRNAs play important roles in PE and have great potential as treatment targets. For clinical application, we propose that miRNA biomarkers combined with traditional biomarkers or miRNA signatures generated from microchips may serve as a great predictive tool for PE occurrence and prognosis. Further, therapies targeting miRNAs or their upstream/downstream molecules need to be developed more quickly to keep up with the progress of routine treatments, such as anticoagulation, thrombolysis, or surgery.

## Introduction

**FIGURE 1:**
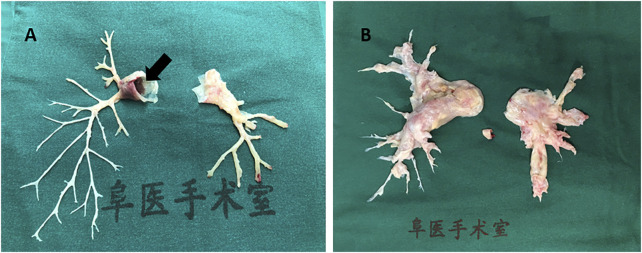
Human pulmonary artery pathological specimen shows **(A)** inflammatory intima specimen accompanied with emboli in pulmonary artery main trunk (black arrow) taken from a patient of pulmonary vasculitis; **(B)** hyperplasia endothelial tissue from pulmonary endarterectomy operation in chronic thrombosis embolic pulmonary hypertension (CTEPH) patient.

Pulmonary embolism (PE) is a common pathologic condition (Doherty, 2017). It is also the third-leading cause of cardiovascular death after cerebral stroke and heart attack (Essien et al., 2019). In particular, massive PE is a life-threatening event with a high mortality rate, and a total of 60,000–100,000 individuals die from PE annually in the United States (Centers for Disease Control and Prevention, 2015). Large pulmonary emboli often impair the hemodynamic condition, leading to high morbidity and mortality. PE is classified into acute PE (APE) and chronic PE (CPE), which may lead to thrombo-embolic pulmonary hypertension (CTEPH). Embolus originating from the lower extremities or pelvis may break apart and spread into the blood stream. A large embolus in the pulmonary circulation can also initiate a coagulation cascade in pulmonary vessels. Recovery abscission of clot fragments can result in CTEPH (Piazza and Goldhaber, 2006). Although multidetector row computed tomography pulmonary angiography improved the understanding of PE as a clinical entity (Doherty, 2017) from 1998 to 2006, the detection rate of PE nearly doubled without any change in its mortality (Long, 2016). Further, although surgical embolectomy has been advocated to treat massive PE, the available medical centers that can perform this complicated surgery are few (Beckerman and Bolotin, 2017). Moreover, due to the compromise of respiration and hemodynamics, a large proportion of patients with acute massive PE do not qualify for surgery. Figure 1 shows a massive embolus retrieved from the pulmonary artery and the chronic hyperplasia pulmonary artery endothelium *via* pulmonary endarterectomy. Deep venous thrombosis (DVT) is the major risk factor of PE. Lower limb extremity deep vein thrombosis is the most likely source of thrombus in PE. Of interest, Badireddy et al. reported that DVT accounts for one-third of all PE cases (Badireddy and Mudipalli, 2021). In recent years, accumulating efforts and attention have been directed to the pathophysiological mechanism of microRNAs (miRNAs), with the aim of developing novel therapies. Further, the novel emerging role of miRNAs as biomarkers contributes to the acceleration of research.

miRNAs are an abundantly expressed subgroup of non-coding RNAs that function as an important endogenous gene regulator, which acts in a post-transcriptional manner. miRNAs regulate the levels of mRNAs for translation by inducing mRNA degradation or translational repression (Bartel, 2004). Currently, approximately 1,000 miRNAs have already been identified, forming an extremely complex miRNA-mRNA regulatory network and strictly controlling nearly every physiological or pathological process. Each miRNA is capable of modulating a specific group of targets, a targetome, and each mRNA might be regulated by multiple miRNAs in a cell type-specific and context-dependent manner (Friedman et al., 2009; Garzon et al., 2009). As a consequence, many disease states are correlated with an abnormal profile of miRNA expression, including cardiovascular, cardiopulmonary, and coagulation conditions, which is quite relevant to PE pathogenesis. This review aimed to summarize the roles of miRNAs in PE as biomarkers and potential novel therapeutic targets and to explore their future research outlook and direction.

## miRNAs as PE Biomarkers

Discovering novel biomarkers for prediction of fatal diseases is a hotspot in medical research. miRNAs have been identified as specific biomarkers, with great potential for clinical diagnosis, especially for diseases wherein protein-based biomarkers are lacking, for example, atrial fibrillation and APE. miRNA profile or signature has a very high sensitivity and specificity for some diseases (Schulte et al., 2017). Circulating miRNA expression is relatively stable in serum, plasma, and whole blood. Cheng et al. showed that these molecules circulate in the vascular system either by being encapsulated in micro-particles/exocrine bodies or binding to protein complexes to avoid degradation (Cheng et al., 2010). As an emerging group of novel non-invasive biomarkers, miRNAs have gradually attracted research attention, and literature has shown that they can be developed as an important biomarker for predicting PE in multiple pathologic events due to their high diagnostic potential (Deng et al., 2016).

### miR-134 and miR-1233

Peng et al. reported that miR-134 and miR-1233 are upregulated 14.59 and 21.56 folds, respectively, in chronic obstructive pulmonary disease (COPD) patients with APE compared to levels in ordinary COPD patients. D-dimer is a classic biomarker for detecting and evaluating PE progression. Of interest, the areas under the curves (AUCs) for the correlation of miR-134 and miR-1233 with PE progression were 0.931 (95% confidence interval [CI] 0.863–0.999) and 0.884 (95% CI 0.79–0.978), respectively, which were higher than those of D-dimer (0.628) (95% CI 0.447–0.809) and Wells score (0.577) (95% CI 0.389–0.765) (Peng et al., 2020). This study provided evidence that the levels of serum miR-1233 and miR-134 are significantly upregulated in COPD patients with APE complication. For miR-134, a meta-analysis also reported that its level was increased in APE patients compared to that in the control group (SMD = 2.84, z = 3.69, *p* < 0.001). The sensitivity, specificity, and diagnostic odds ratio were 0.86 (0.72–0.94), 0.75 (0.66–0.82), and 19 (7–51) respectively, which indicated its great potential as a PE biomarker (Liu et al., 2021). Among the variety of miRNA-related PE studies, research on miR-134 is the most extensive and reliable up to date (Xiao et al., 2011; Xiang et al., 2019). For miR-1233, it was reported to be the first miRNA biomarker for distinguishing APE from acute non-ST elevated myocardial infarction and healthy individuals (Kessler et al., 2016). Considering the confusing and overlapping clinical symptoms between APE and myocardial infarction, we consider these findings as exciting and meaningful evidences that contribute to the field.

### miR-532-5p and miR-483-3p

miR-483-3p and miR-532-5p were found to correlate with PE, with sensitivity, specificity, and AUC of (1.00, 1.00, and 1.00) and (1.00, 1.00, and 1.00), respectively (Xiang et al., 2019). From the mechanistic point of view, miR-532-5p and miR-483-3p were found to share the same target, NFATC2IP gene, which is expressed in T-helper two cells and regulates the transcription of cytokine genes, including IL-3, 4, 5 and 13. In patients with PE, cytokines are abnormally expressed in peripheral circulating mononuclear cells. Moreover, the cellular immune response and pathways are impaired in PE, which is highly relevant to cytokine expression and implies the correlation between PE/venous thrombosis embolism and cellular immune dysfunction (Lv et al., 2013). Further, the target genes of these two miRNAs include molecules in the MAPK and PI3K-Akt signaling pathways, which are well-known to be involved in macrophage inflammatory, oxidative stress response (Zhang et al., 2015) as well as the apoptosis process of endothelial cells (Lou et al., 2016).

### miR-221 and miR-27a/B

Liu et al. reported**,** in a cohort of 60 PE patients and 50 healthy volunteers, a significant increase in miR-221 level in the plasma from APE patients compared to that in healthy individuals. Correlation analysis showed a positive correlation between this miRNA and BNP (r = 0.842, *p* < 0.05), troponin I (r = 0.853; *p* < 0.05), and D-dimer (r = 0.838; *p* < 0.05) (Liu et al., 2018). Wang et al. also found that plasma miR-27a/b levels were significantly higher and showed positive correlation in APE patients compared to in the control group. Receiver operating characteristic (ROC) curve analyses showed that plasma miR-27a was superior to miR-27b with regard to the diagnosis of APE (AUC = 0.784, AUC = 0.707, respectively). Combining miR-27a or miR-27b with D-dimer significantly increased the diagnostic capacity for APE (Wang et al., 2018). Furthermore, miR-27a/b is related to the proliferation of pulmonary artery smooth muscle cells (PASMC). Endothelin-1 (ET-1) can stimulate miR-27a/b expression by activating the NF-κB pathway, which subsequently leads to a decrease in peroxisome proliferator-activated receptor γ and contributes to ET-1-induced PASMC proliferation. These processes may have a correlation with pulmonary vascular remodeling and pulmonary hypertension (Xie et al., 2017).

### miR-338-5p

This miRNA has been proved to play a role in virus-associated cancers, such as esophageal squamous cell carcinoma, and neuropathies, such as Alzheimer’s disease (Han et al., 2019; Qian et al., 2019). A recent publication by Zhang et al. reported that inhibition of miR-338-5p can increase the level of IL-6, which subsequently promotes the development and progression of DVT in a mouse study. In a cell model, it was shown that overexpression of miR-338-5p downregulated the expression of IL-6, while miR-338-5p inhibition upregulated IL-6 expression. An *in vivo* study found that mice treated with anti-IL-6 antibody or agomiR-338-5p delivery exhibited decreased expression of IL-6 and alleviation of DVT, whereas antagomiR-338-5p aggravated the progression of DVT (Zhang et al., 2020a).

### miR-28-3p

Zhou et al. reported that among the twelve miRNAs they studied, miR-28-3p showed a significant increase in the plasma from PE patients. However, the increase in miR-28-3p level due to PE development was not significant compared to its level in the experimental dogs before inducing PE. The AUC of the ROC of plasma miR-28-3p was 0.792 (95% CI: 0.689–0.896). Moreover, pathway analysis revealed that the effect of miR-28-3p involves inositol phosphate metabolism and the phosphatidylinositol signaling system, which are highly relevant pathways to PE pathogenesis (Zhou et al., 2016).

The circulating miRNAs as biomarkers of PE introduced above may not be comprehensive, and there should be many other potential miRNAs for predicting PE (Suzuki et al., 2016). Using only one miRNA as a biomarker may have limitations. In the future, it is possible to use a group of miRNAs to achieve the ideal sensitivity and specificity for PE biomarker assays.

## miRNAs are Potential Novel Therapeutic Targets for PE

Regarding APE treatment, one of the classic approaches is to inhibit the excessive proliferation and migration of PASMCs. Several miRNAs have been characterized and validated to regulate cell proliferation, apoptosis, and other related physiological processes. Therefore, finding miRNAs specific to these regulatory mechanisms may provide future therapeutic targets for PE or pulmonary artery hypertension induced by acute PE (APE-PAH).

### miR-340-5p

A recent study on APE-PAH found that in APE-PAH patients, miR-340-5p was lowly expressed, whereas IL-1β and IL-6 were highly expressed. In a large sample cohort, linear regression was found negatively correlated between miR-340-5p and IL-1β/IL-6. In cell culture, upon miR-340-5p overexpression, the levels of IL-1β and IL-6 were reduced, coupled with decreased proliferation and migration of PASMCs as well as ameliorated inflammatory response. As is well known, IL-1 has been characterized to significantly induce IL-6 expression (Tosato and Jones, 1990). In the dual-luciferase reporter assay regarding miR-340-5p, IL-1β and IL-6, both pro-inflammatory targets were confirmed to be downregulated by miR-340-5p directly, revealing a synergistic anti-inflammation effect of miR-340-5p. Therefore, miR-340-5p might be a promising therapeutic target as an anti-inflammatory agent (Ou et al., 2020).

### miR-160b-5p

In an APE mouse model, Chen et al. demonstrated miR-106-5p as a novel regulator of PASMC proliferation and pulmonary vascular remodeling, and it performed its functions *via* targeting neuron-derived orphan receptor-1 (NOR1). Several literatures have reported NOR1 to regulate vascular cell migration and proliferation, and these important cellular processes are highly correlated with inflammation, growth factors, lipoproteins, and thrombin in vascular diseases (Martínez-González et al., 2003; Rius et al., 2004). Importantly, these findings highlight a novel therapeutic mechanism and improve the biological understanding of the complex network modulating APE progression (Chen et al., 2020).

### miR-22-3p

Yang et al. reported that resveratrol successfully prevented PE-induced cardiac injury, possibly by modulating metastasis-associated lung carcinoma transcript 1 (MALAT1) expression through promoter regulation. MALAT1 was found to directly target miR-22-3p, as revealed by luciferase assay. Furthermore, miR-22-3p can bind complementarily to the 3′ UTR of NLR family pyrin domain containing 3 (NLRP3), leading to downregulation of NLRP3. In PE-associated cardiac injury, it was found that the levels of MALAT1, NLRP3, caspase-1, IL-1β, and IL-18 were significantly elevated, while miR-22-3p level was downregulated (Yang et al., 2019).

### miR-21

A very recent study reported that curcumin can improve lung edema and outcomes of APE rats and that it decreased mPAP and RVSP levels, wet/dry weight ratio, thrombus size, and inflammatory markers in lung tissues. The possible mechanism is that curcumin modulated the miR-21-Sp1-PTEN axis, which reduced the NF-κB signaling pathway, thus suppressing lung injury and inflammation in APE (Liang et al., 2021).

### miRNAs Correlated With Matrix Metalloproteinases and Venous Thrombophilia

As is known, pulmonary embolism is highly related to matrix metalloproteinases (MMPs) and venous thrombus (VT). miRNAs that modulate MMP biogenesis/degradation balance or VT pathophysiology have been demonstrated to indirectly modulate outcome of APE (Goldhaber and Bounameaux, 2012; Neto-Neves et al., 2013b).

Aberrant MMP activity has been demonstrated to be associated with APE-induced hemodynamic dysregulation. Neto-Neves et al. reported that MMPs were lowly expressed in healthy lung tissues and that the expression and activity of MMPs were augmented in lung injury (Neto-Neves et al., 2013a). Doxycycline, an inhibitor of MMP activity, was found to improve APE prognosis and protect against RV enlargement in a rat model, possibly *via* amelioration of APE-induced oxidative stress and inhibition of ventricular proteolytic activity (Cau et al., 2013). In addition, l-arginine also attenuated APE-induced pulmonary hypertension through other mechanisms, including the modulation of NO synthesis and downregulation of MMP-2 and MMP-9 activities (Souza-Costa et al., 2005). Therefore, specific miRNAs that are capable of modulating MMP expression also have potential treatment or prediction value in PE pathogenesis. Zhang et al. elucidated that miR-15a-5p expression was significantly increased in the lung tissue of PAH rats and that artificial overexpression of miR-15a-5p led to increased expression of MMP-2 (Zhang et al., 2020b). Some studies found that inhibition or degradation of the corresponding mRNA of MMP-12 could be a solution to treat pathological lung tissue remodeling (Garbacki et al., 2009).

VT is a complex pathologic condition with a highly heritable genetic component, which predisposes an individual to its development. VT often induces the occurrence of thrombosis or embolism, including PE, in multiple sites in both veins and arteries (Sun et al., 2021). There are four miRNAs (hsa-miR-126-3p, hsa-miR-885-5p, hsa-miR-194-5p, and hsa-miR-192-5p) that have been studied and proved to be correlated with VT. These showed significant correlations with intermediate phenotypes of VT. It was found that multiple miRNAs were correlated with several coagulation-related factors: miR-885-5p and miR-195-5p with the level of protein S and FVII; miR-192-5p with FVII and ADAMS13; and miR-126-3p with factor XI (Rodriguez-Rius et al., 2020). miR-494 has been shown to downregulate the expression of PROS1 and protein S in liver Huh cells (Tay et al., 2013). Further, factor X deficiency was found to be associated with aberrant expression of miR-24. In a cohort of 15 healthy adults and 36 severe trauma-induced coagulopathy patients, plasma miRNA screening showed that all coagulopathy patients had elevated levels of miR-24 and lower levels of factor X than did the healthy volunteers (Chen et al., 2017). It is believed that the expression pattern of miRNAs that target coagulation factors can result in thrombosis and coagulation-related diseases (Jankowska et al., 2020).

## microRNA and its Research Outlook in PE

With the popularization of sequencing and development of multiple human genetic projects, nearly 1,000 miRNAs have been identified, and the miRNA group has been predicted to regulate 30% of all human transcripts. Despite the accumulating literature and rapid progress in miRNA studies and research tools, only a small proportion of miRNAs have been well-characterized in dedicated cell types or diseases (Chang and Mendell, 2007). Nevertheless, they have been proved to have various functions that correlate with cancer, metabolic disease, neural system disease, and cardiovascular disease (Iorio and Croce, 2012; Vienberg et al., 2017; Wojciechowska et al., 2017; Juźwik et al., 2019; Hembrom et al., 2020). Microarrays are often used to find the correlations between miRNAs and diseases. A microarray study by Guo et al. revealed differential miRNA profile in CTEPH patients compared to that in healthy individuals, providing fundamental data for pathologic studies and biomarker screening for CTEPH. It was reported that a reduced level of let-7b might be involved in the pathogenesis of CTEPH by modulating ET-1 expression in pulmonary artery endothelial cells and PASMCs (Guo et al., 2014). In-silico studies have provided information on miRNAs that participate in the etiology of venous thrombosis, including their specific targetnetwork, and further investigation with a larger sample cohort is needed to explore and validate the mechanism and functional nature of miRNAs.

Further, as biomarkers for PE, miRNAs and their microchips may play more important roles in predicting PE or pre-judging PE prognosis. However, the existing biomarkers, such as D-dimer, are still necessary. Combining miRNAs and traditional biomarker molecules may increase their specificity and sensitivity. For example, studies have reported that accurate prediction of DVT can be achieved by comprehensive co-analysis of miR-96 and D-dimer level in patient plasma samples (Xie et al., 2016). Moreover, Jiang et al. reported that detection and co-analysis of miR-320a/b and D-dimer might further improve the accuracy of DVT diagnosis (Jiang et al., 2018). Because of the increasing number of miRNA research and complex network of PE biomarkers with their targets, Table 1 has been provided to briefly display and visualize them.

**TABLE 1:** microRNAs’ function and their characteristics from literature.

microRNA	Sensitivity	Specificity	AUC
microRNA-134	0.86	0.75	0.931
microRNA-1233	0.89	0.95	0.940
microRNA-532	1.00	1.00	1.000
microRNA-483-3p	1.00	1.00	1.000
microRNA-27a	0.78	0.83	0.784
microRNA-27b	0.65	0.78	0.707
microRNA-221	N.A	N.A	0.823
microRNA-28-3p	0.84	0.81	0.792
microRNA-22	0.63	0.79	0.750
let-7b	0.87	0.60	0.770

AUC, area under curve; N.A, not available.

In view of integrating miRNA observational study and functional analysis, there are still limitations and gap between miRNA as biomarker and miRNA as potential therapeutic method. Compared to biomarkers which correlate its expression with disease phenotype, miRNA therapeutics need further fundamental experiments to validate and decipher its underlying mechanism. As for current miRNA therapeutic clinical trials, the majority is focused on liver or circulation, by an approach of systemic injection (Zhang et al., 2021). A recent miRNA phase I study used MRX34, a liposomal miR-34a mimic to treat advanced solid tumors. The results showed a manageable toxicity level and improved clinical outcome (Hong et al., 2020). However, there is still long way to march in order of pushing miRNA from biomarker, animal study into reality treatment.

## Conclusion

This review focused on the functions of miRNAs in PE. As the increasing number of miRNA and its functions being studied in PE, the predictive and therapeutic value of miRNA has been gradually improving. However, physicians do not see the importance of miRNAs in their clinical practice because they still use traditional biomarkers, such as D-dimer, and anti-coagulation agents or thrombolysis drugs. We need to break the barrier between scientific research and the clinical application of miRNAs in the future. The development of accurate and highly sensitive miRNA microchips that can predict APE or CTEPH using blood may be a future direction. We also expect that assays that combine miRNAs and traditional biomarkers of PE can be developed in the future. Further, because miRNAs function in gene expression, novel effective drugs, which can prevent or treat PE more efficiently than the current old and routine methods, such as anti-coagulation, thrombolysis, and surgery, are expected to be developed. So far, only literature on miRNAs exists, which cannot affect the diagnosis and treatment of diseases. We are thus eager to see more actual products that can be used in clinical practice in the near future.
