# Corrigendum: The Function of microRNAs in Pulmonary Embolism: Review and Research Outlook

**DOI:** 10.3389/fphar.2021.822059

**Published:** 2022-01-05

**Authors:** Mingyao Luo, Mingyuan Du, Chang Shu, Sheng Liu, Jiehua Li, Lei Zhang, Xin Li

**Affiliations:** ^1^ State Key Laboratory of Cardiovascular Diseases, Center of Vascular Surgery, Fuwai Hospital, National Center for Cardiovascular Diseases, Chinese Academy of Medical Science and Peking Union Medical College, Beijing, China; ^2^ Department of Vascular Surgery, The Second Xiangya Hospital, Central South University, Changsha, China; ^3^ The Institute of Vascular Diseases, Central South University, Changsha, China

**Keywords:** biomarker, deep venous thrombosis, miRNA, molecular regulation, pulmonary embolism, treatment target

In the original article, there was a mistake in the caption for Figure 1A as published. The corrected sentence in the caption reads as follows: “**(A)** inflammatory intima specimen accompanied with emboli in pulmonary artery main trunk (black arrow) taken from a patient of pulmonary vasculitis.” The corrected Figure 1 appears below.

**FIGURE 1:**
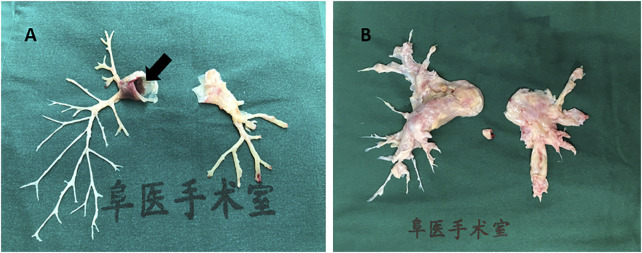
Human pulmonary artery pathological specimen shows **(A)** inflammatory intima specimen accompanied with emboli in pulmonary artery main trunk (black arrow) taken from a patient of pulmonary vasculitis; **(B)** hyperplasia endothelial tissue from pulmonary endarterectomy operation in chronic thrombosis embolic pulmonary hypertension (CTEPH) patient.

The authors apologize for this error and state that it does not change the scientific conclusions of the article in any way. The original article has been updated.

